# Crucial role of RAGE in inappropriate increase of smooth muscle cells from patients with pulmonary arterial hypertension

**DOI:** 10.1371/journal.pone.0203046

**Published:** 2018-09-04

**Authors:** Kazufumi Nakamura, Masakiyo Sakaguchi, Hiromi Matsubara, Satoshi Akagi, Toshihiro Sarashina, Kentaro Ejiri, Kaoru Akazawa, Megumi Kondo, Koji Nakagawa, Masashi Yoshida, Toru Miyoshi, Takeshi Ogo, Takahiro Oto, Shinichi Toyooka, Yuichiro Higashimoto, Kei Fukami, Hiroshi Ito

**Affiliations:** 1 Department of Cardiovascular Medicine, Okayama University Graduate School of Medicine, Dentistry and Pharmaceutical Sciences, Okayama, Japan; 2 Department of Cell Biology, Okayama University Graduate School of Medicine, Dentistry and Pharmaceutical Sciences, Kita-ku, Okayama, Japan; 3 Division of Cardiology, National Hospital Organization Okayama Medical Center, Okayama, Japan; 4 Division of Advanced Medicine for Pulmonary Hypertension, Division of Pulmonary Circulation, Department of Cardiovascular Medicine, National Cerebral and Cardiovascular Centre, Osaka, Japan; 5 Departments of General Thoracic, Breast and Endocrinological Surgery, Okayama University Graduate School of Medicine, Dentistry and Pharmaceutical Sciences, Okayama, Japan; 6 Department of Medical Biochemistry, Kurume University School of Medicine, Kurume, Japan; 7 Division of Nephrology, Department of Medicine, Kurume University School of Medicine, Kurume, Japan; University of Alabama at Birmingham, UNITED STATES

## Abstract

**Background:**

Pulmonary vascular remodeling of pulmonary arterial hypertension (PAH) is characterized by an inappropriate increase of vascular cells. The receptor for advanced glycation end products (RAGE) is a type I single-pass transmembrane protein belonging to the immunoglobulin superfamily and is involved in a broad range of hyperproliferative diseases. RAGE is also implicated in the etiology of PAH and is overexpressed in pulmonary artery smooth muscle cells (PASMCs) in patients with PAH. We examined the role of RAGE in the inappropriate increase of PASMCs in patients with PAH.

**Methods and results:**

PASMCs were obtained from 12 patients with PAH including 9 patients with idiopathic PAH (IPAH) and 3 patients with heritable PAH (HPAH) (2 patients with *BMPR2* mutation and one patient with *SMAD9* mutation) who underwent lung transplantation. Western blot analysis and immunofluorescence staining revealed that RAGE and S100A8 and A9, ligands of RAGE, were overexpressed in IPAH and HPAH-PASMCs in the absence of any external growth stimulus. PDGF-BB (10 ng/mL) up-regulated the expression of RAGE in IPAH and HPAH-PASMCs. PAH-PASMCs are hyperplastic in the absence of any external growth stimulus as assessed by 3H-thymidine incorporation. This result indicates overgrowth characterized by continued growth under a condition of no growth stimulation in PAH-PASMCs. PDGF-BB stimulation caused a higher growth rate of PAH-PASMCs than that of non-PAH-PASMCs. AS-1, an inhibitor of TIR domain-mediated RAGE signaling, significantly inhibited overgrowth characterized by continued growth under a condition of no growth stimulation in IPAH and HPAH-PASMCs (P<0.0001). Furthermore, AS-1 significantly inhibited PDGF-stimulated proliferation of IPAH and HPAH-PASMCs (P<0.0001).

**Conclusions:**

RAGE plays a crucial role in the inappropriate increase of PAH-PASMCs. Inhibition of RAGE signaling may be a new therapeutic strategy for PAH.

## Introduction

Pulmonary arterial hypertension (PAH) is a progressive disease characterized by elevated levels of pulmonary artery pressure (PAP) and pulmonary vascular resistance (PVR). Increased PVR is caused by pulmonary vasoconstriction and by vascular remodeling and thrombosis [1, 2]. Pulmonary vascular remodeling is caused by an inappropriate increase of vascular cells including pulmonary artery smooth muscle cells (PASMCs) [3]. In order to archive reverse remodeling of pulmonary vessels, anti-proliferative agents for PASMCs are required. Treatment of pulmonary hypertension has progressed due to the recent development of pulmonary arterial hypertension-targeted drugs. However, long-term survival of patients with idiopathic PAH (IPAH) and heritable PAH (HPAH) is still suboptimal. Therefore, new treatment that archives reverse remodeling is thought to be needed [4].

The receptor for advanced glycation end products (RAGE) is a type I single-pass transmembrane protein belonging to the immunoglobulin superfamily [5]. RAGE is thought to be involved in a broad range of inflammatory, degenerative and hyperproliferative diseases, including sepsis, rheumatoid arthritis, diabetic nephropathy, atherosclerosis and cancer [6]. RAGE is highly expressed in the lung and is associated with the pathogenesis of asthma [7, 8]. RAGE is also implicated in PAH etiology [9]. RAGE is overexpressed in PASMCs of patients with PAH. RAGE inhibition by siRNA decreases proliferation of PASMCs and shows therapeutic effects in monocrotaline- and sugen-induced PAH rats [9].

The aim of this study was to determine the role of RAGE in the inappropriate increase of PASMCs in patients with PAH including IPAH and HPAH. We examined 1) whether expression levels of RAGE and S100A8/A9, ligands of RAGE, were elevated in PAH-PASMCs, 2) whether platelet-derived growth factor (PDGF)-BB, an intense growth factor of PASMCs, up-regulated the expression of RAGE and 3) whether AS-1, an inhibitor of RAGE signaling, has anti-proliferative effects on PAH-PASMCs.

## Materials and methods

### Isolation, culture and identification of PASMCs

Peripheral segments of the pulmonary artery were obtained at lung transplantation from 12 patients with PAH as previously described [10–12] (3 males and 9 females; mean age, 19±11 years; age range, 8–43 years) (Table 1). For normal control experiments, samples of pulmonary arteries were also obtained at lung lobectomy from 9 patients with bronchogenic carcinoma (6 males and 3 females; mean age, 55±17 years; age range, 28–76 years) who showed no evidence of PAH and did not receive any systemic chemotherapy or radiation therapy before lung lobectomy as previously described [10, 11]. Samples of the pulmonary arteries were obtained from the most distal area from the carcinoma in the resected lobe. All of the studies were approved by the Ethics Committee of Okayama University Graduate School of Medicine, Dentistry, and Pharmaceutical Sciences, and written informed consent was obtained from all patients before the procedure.

**Table 1:** Clinical data of patients with PAH.

Patient	Age	Sex	Diagnosis	Gene mutation	PAP (s/d/m)	CI	PVR
	(years old)			(Gene, amino acid change)	(mmHg)	(L/min/m^2^)	(dyne·sec·cm^–5^)
1	31	F	IPAH		73/30/48	2.1	1199
2	13	F	IPAH		111/49/67	1.7	2438
3	28	F	IPAH		113/36/66	1.8	3340
4	43	F	IPAH		107/47/72	2.4	3056
5	16	F	IPAH		83/51/65	2.5	784
6	11	M	IPAH		130/51/80	1.9	2629
7	8	F	IPAH		98/54/74	3.1	1784
8	10	F	IPAH		60/35/45	2.8	1040
9	25	M	IPAH		77/35/50	2.8	532
10	12	F	HPAH	*SMAD9*, p.Lys43Glu	99/59/72	2.3	2779
11	20	M	HPAH	*BMPR2*, p.Leu139Ser	70/40/50	3.3	808
12	11	F	HPAH	*BMPR2*, p.Arg491Gln	106/47/67	3.2	1148

PAH: pulmonary arterial hypertension, PAP: pulmonary artery pressure, s/d/m: systolic/diastolic/mean, CI: cardiac index, PVR: pulmonary vascular resistance, F: female, M: male, IPAH: idiopathic pulmonary arterial hypertension, HPAH: heritable pulmonary arterial hypertension.

PASMCs were isolated as described previously [10, 11]. Peripheral segments of pulmonary arteries smaller than 1 mm in outer diameter were disaggregated with collagenase and cut into 2-mm-long sections, and then the adventitia layer was removed. The segments of pulmonary arteries were plated on a 6-well plate with Dulbecco’s modified Eagle’s medium (DMEM; Gibco, Grand Island, NY, USA) supplemented with 10% fetal bovine serum (FBS; Sigma) and 0.1 mg/mL kanamycin (Sigma) and incubated in a humidified 5% CO2 atmosphere at 37°C. The culture medium was changed every 3 days. After reaching confluence, the cells were subcultured by treatment with trypsin (0.05%)/ethylenediaminetetraacetic acid (EDTA) (0.02%). Cell identification was confirmed by examination of cytoskeletal components, α-smooth muscle actin (αSMA), myosin, and smoothelin, using an immunocytochemical technique as described previously [10, 11]. Cells between passages 3 to 5 were used for all experiments.

### Immunofluorescence staining of RAGE, S100A8 and S100A9

Immunohistochemical and immunocytochemical analyses were performed to confirm the expression of RAGE, S100A8 and S100A9 in sections of lungs and in PASMCs. For immunohistochemical analysis, lungs from patients with PAH or control subjects were fixed with 4% paraformaldehyde. Immunofluorescence was performed on 5-μm lung slices. For immunocytochemical analysis, PASMCs were fixed with 4% paraformaldehyde and permeabilized with 0.2% Triton X-100. Rabbit anti-human RAGE antibody (Santa Cruz Biotechnology), mouse anti-αSMA antibody (Sigma-Aldrich), goat anti-S100A8 (calgranulin A) antibody (Santa Cruz Biotechnology) and goat anti-S100A9 (calgranulin B) antibody (Santa Cruz Biotechnology) were used. The second antibodies were swine anti-rabbit (FITC, F0205) (Dako), rabbit anti-mouse (TRITC, R0270) (Dako) and rabbit, anti-goat (FITC, F0250) (Dako).

### Western blot analysis

PASMCs were reseeded in 24-well plates at a density of 5 x 10^4^ cells/well on day 0. After 16 hours of incubation (on day 1), the culture media were replaced with low-serum culture media (DMEM, 0.1% FBS, and 0.1 mg/mL kanamycin), and the cultured cells were made quiescent for 48 hours. On day 3, PDGF-BB (10 ng/mL) (Sigma) or its diluent (phosphate buffered saline containing 0.1% bovine serum albumin) was added to the media. After 24 hours (on day 4), total cell lysates of cultured PASMCs were extracted in commonly used radioimmunoprecipitation (RIPA) buffer with 10 mg/mL phenylmethylsulfonyl fluoride (Sigma) and then concentrated by centrifugation at 12,000 rpm for 20 minutes.

Western blot analysis was performed as described previously [6, 13]. Rabbit anti-human RAGE antibody (A-9) (Santa Cruz Biotechnology) and anti-tubulin antibody (mouse anti-human tubulin antibody (Sigma-Aldrich) were used. The second antibody was horseradish peroxidase-conjugated anti-mouse or anti-rabbit IgG antibody (Cell Signaling Technology). Positive signals were detected by a chemiluminescence system (ECL plus, GE Healthcare Bio-Sciences, Piscataway, NJ).

### Cell proliferation assay

To assess the antiproliferative effect of AS-1 or RAGE aptamer on PASMCs, we measured 3H-thymidine incorporation using methods described previously [3, 10]. We previously reported that PDGF-BB (10 ng/mL) stimulation causes a higher growth rate of cultured PASMCs from patients with PAH than that of control cells [10, 14, 15]. PASMCs were reseeded in 24-well plates at a density of 5 x 10^4^ cells/well on day 0. After 16 hours of incubation (on day 1), the culture media were replaced with low-serum culture media (DMEM, 0.1% FBS, and 0.1 mg/mL kanamycin), and the cultured cells were made quiescent for 48 hours. On day 3, PDGF-BB (10 ng/mL) (Sigma), its diluent (phosphate buffered saline containing 0.1% bovine serum albumin), AS-1 (100 μmol/L) (Calbiochem), a RAGE aptamer (100 nmol/L) or control aptamer (100 nmol/L) was added to the media. After 21 hours (on day 4), the cells were labeled with ^3^H-thymidine at 1 μCi/mL for 3 hours. After completion of labeling, the cells were washed with ice-cold PBS, fixed with 5% trichloroacetic acid and 95% ethanol, and lysed with 200 μL/well of 0.33 mol/L NaOH. Aliquots of the cell lysates were neutralized with 1 mol/L HCl, and the radioactivity was measured in a liquid scintillation analyzer (TRI-CARB 2200CA; Packard, Downers Grove, IL, USA).

### DNA-aptamer directed against RAGE (RAGE aptamer)

RAGE aptamer was obtained by an in vitro selection process, that is, SELEX method, from a pool of ~10^15^ different nucleic acid sequences as previously described [16–18]. Sequences directed against RAGE were obtained in this study, and sequences of the selected RAGE and control aptamers are “cATTcTTAgATTTTTgTcTcAcTTAggTgTAgATggTgAT” and “aTcgAccTggAggcgAgcAgcTcggATccAgTcgcgTgAg”, respectively. These aptamers were modified with phosphorothioate for protection against degradation by nuclease." Phosphorothioate nucleotides are indicated by capital letters.

### Statistical analysis

Data are expressed as mean ± standard deviation (SD). Statistical significance for comparison between two measurements was determined using Student’s t-test. For comparison between different treatment groups, statistical analysis was performed using one-way ANOVA with Fisher’s protected least significant difference test. Values of P < 0.05 were considered to be significant.

## Results

### RAGE and S100A8/A9 expression in PASMCs of patients with PAH

**Fig 1:**
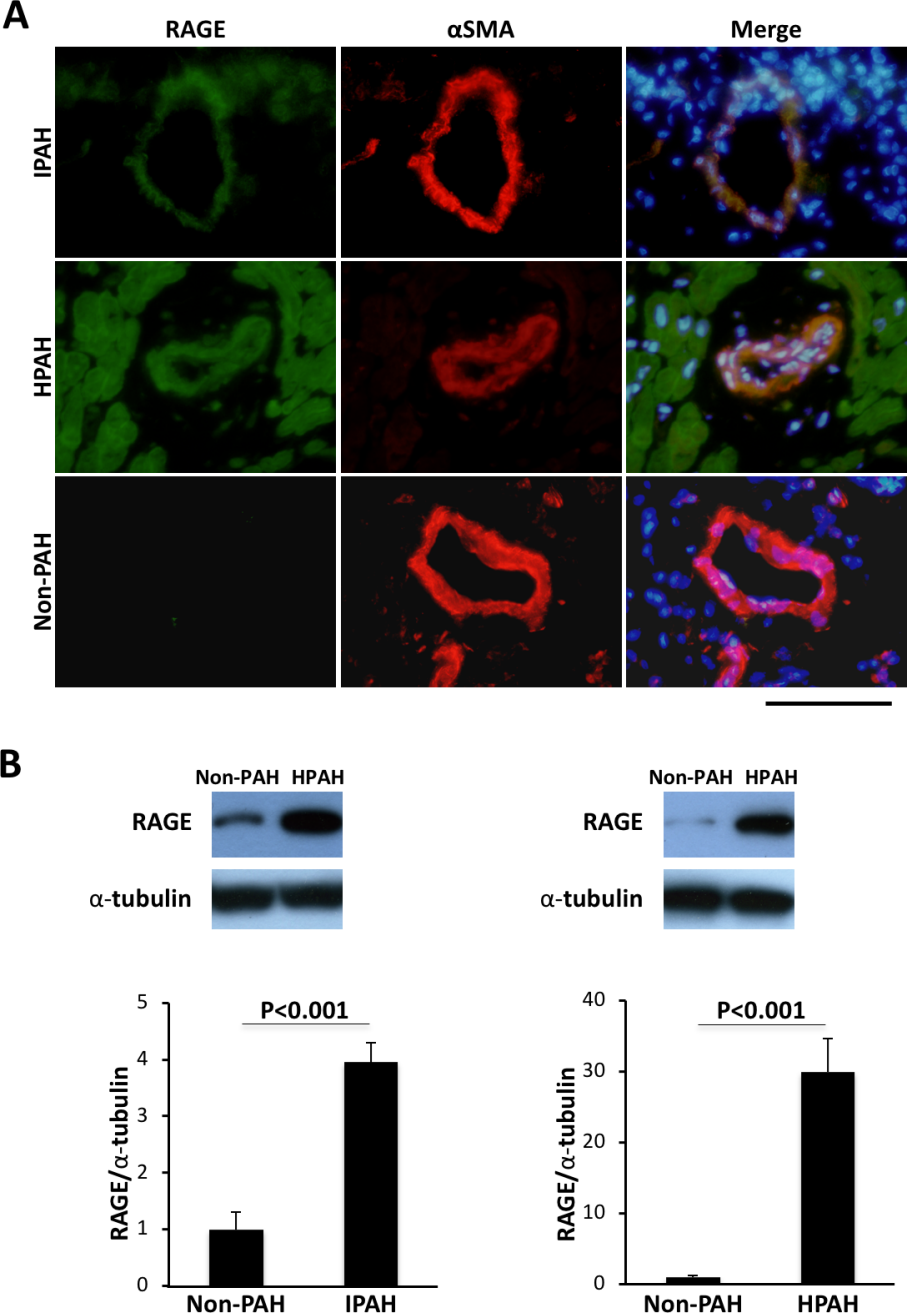
RAGE expression in PASMCs of patients with PAH. A. Immunohistochemical staining of RAGE (green) (left figures), α-SMA (red) (center figures) and merge images (right figures) in distal pulmonary arteries of patients with IPAH (upper figures), patients with HPAH (middle figures) and non-PAH patients (lower figures). Nuclear staining was performed by using DAPI (blue). Bar = 100 μm. B. Western blot analysis of RAGE in PASMCs of patients without PAH (non-PAH) versus patients with IPAH (left) and patients without PAH (non-PAH) versus patients with HPAH (right). Data are mean ± SD.

Firstly, we examined whether expression levels of RAGE and S100A8/A9, ligands of RAGE, were elevated in PASMCs of patients with PAH. Immunohistochemical analysis revealed that RAGE was expressed in PASMCs of distal pulmonary arteries from patients with PAH, but RAGE was not expressed in those of patients without PAH (Fig 1A). Western blot analysis also revealed that expression levels of RAGE in IPAH and HPAH-PASMCs in the absence of any external growth stimulus condition were significantly higher than the expression levels in non-PAH-PASMCs (Fig 1B).

**Fig 2:**
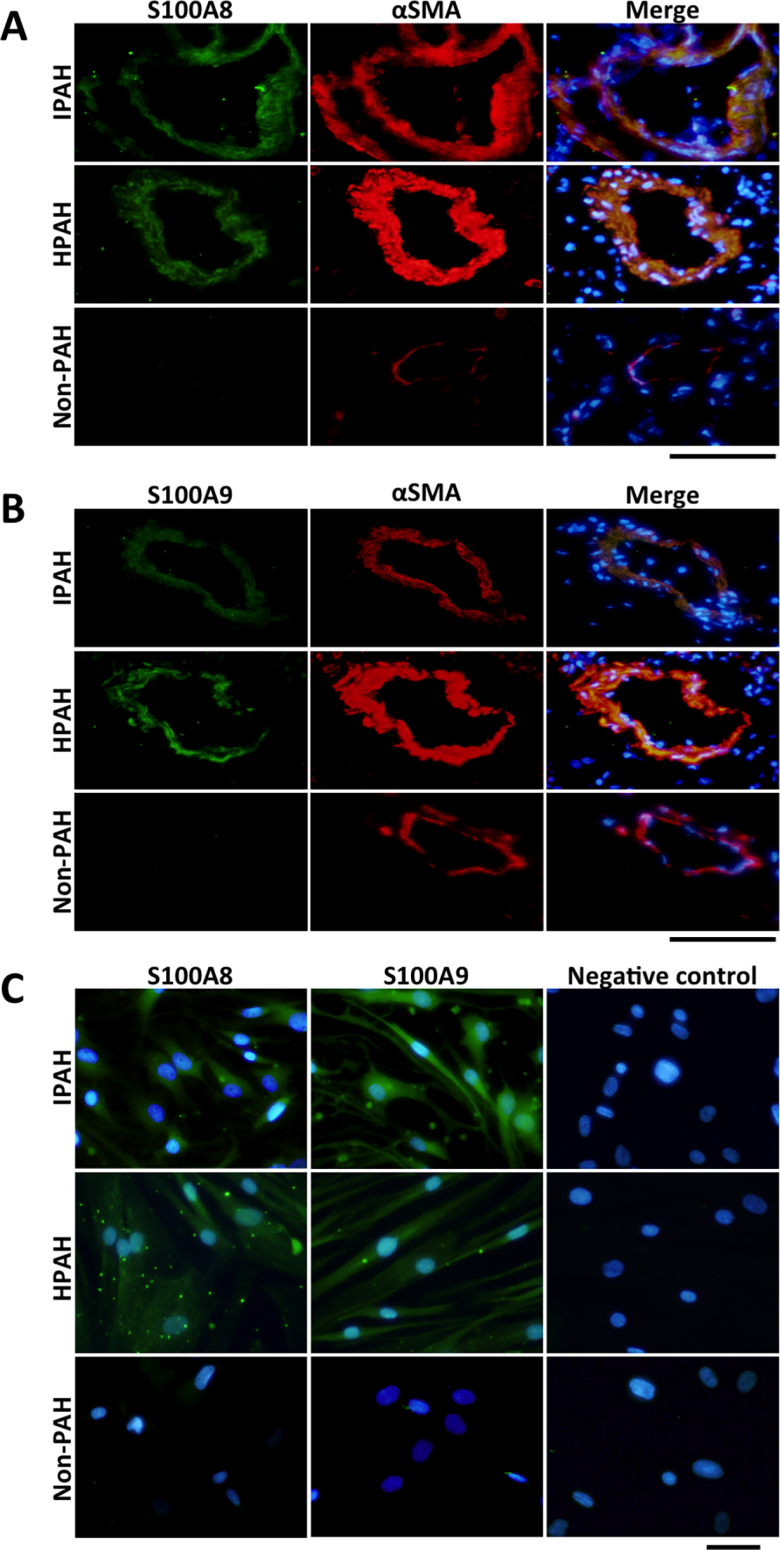
S100A8/A9 expression in PASMCs of patients with PAH. A. Immunohistochemical staining of S100A8 (green) (left figures), α-SMA (red) (center figures) and merge images (right figures) in distal pulmonary arteries of patients with IPAH (upper figures), patients with HPAH (middle figures) and without PAH (non-PAH) (lower figures). B. Immunohistochemical staining of S100A9 (green) (left figures), α-SMA (red) (center figures) and merge images (right figures) in distal pulmonary arteries of patients with IPAH (upper figures), patients with HPAH (middle figures) and patients without PAH (non-PAH) (lower figures). C. Immunocytochemical analysis of S100A8 (green) (left figures), S100A9 (green) (center figures) and negative control staining without 1^st^ antibody (left figures) in cultured PASMCs of patients with IPAH (upper figures), patients with HPAH (middle figures) and patients without PAH (non-PAH) (lower figures). Nuclear staining was performed by use of DAPI (blue). Bar = 100 μm.

S100A8/A9 were also expressed in PASMCs of distal pulmonary arteries from patients with IPAH and HPAH, but they were not expressed in those of patients without PAH as assessed by immunohistochemical analysis (Fig 2A and 2B). S100A8/A9 were observed in three serial sections from patients with IPAH, HPAH and non-PAH. S100A8/A9 were also expressed in cultured PASMCs obtained from patients with IPAH and HPAH in the absence of any external growth stimulus condition, but they were not expressed in cultured PASMCs obtained from patients without PAH under the same condition (Fig 2C). These results indicate that RAGE and S100A8/A9 were overexpressed in PAH-PASMCs in the absence of any external growth stimulus.

### Up-regulation of the expression of RAGE in PASMCs of patients with PAH by PDGF-BB stimulation

**Fig 3:**
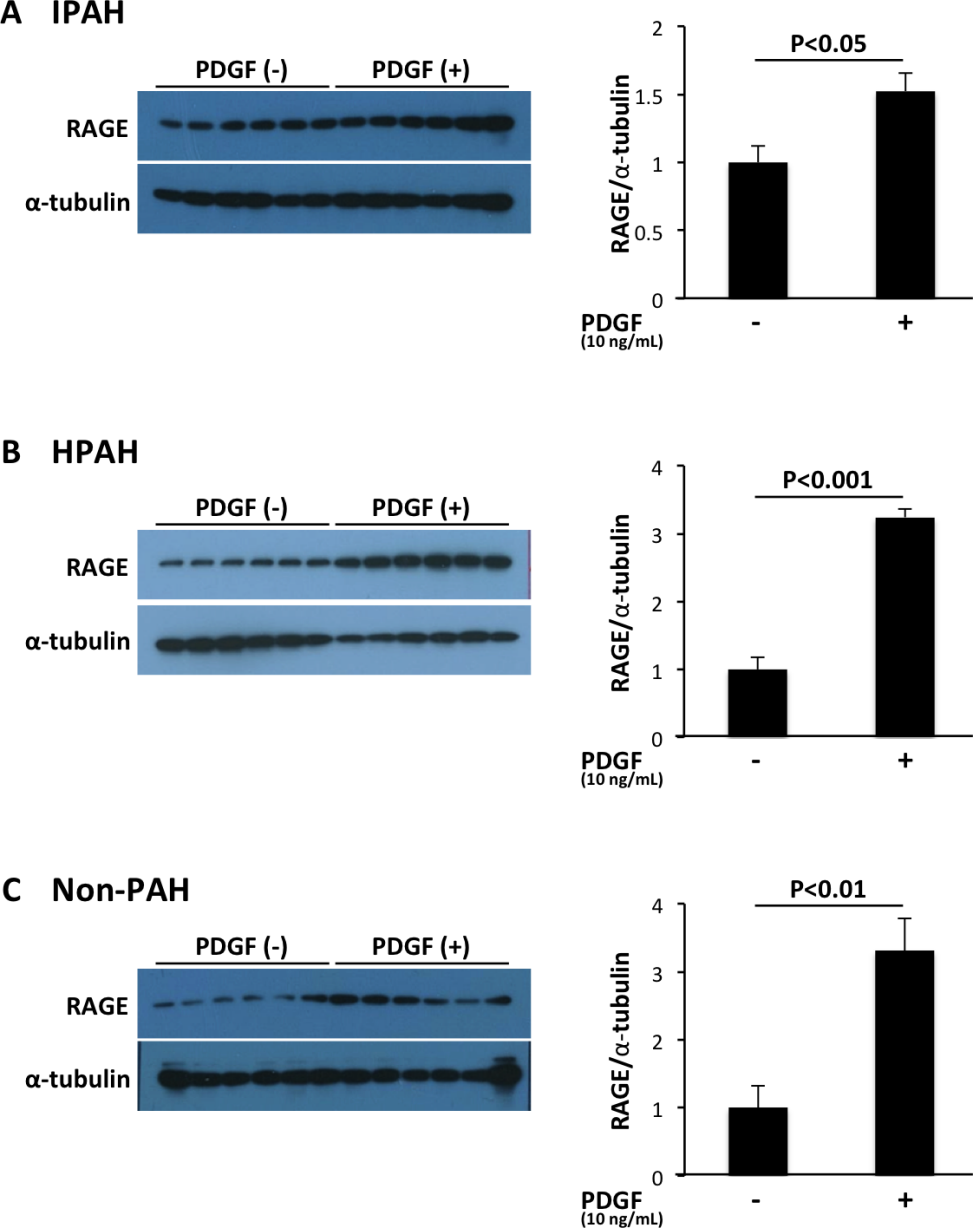
Increased expression of RAGE in PASMCs of patients with PAH by PDGF-BB stimulation assessed by western blot analysis. PDGF-BB (10 ng/mL) up-regulated the expression of RAGE in PASMCs of patients with IPAH (A), patients with HPAH (B) and patients without PAH (non-PAH) (C). Data are mean ± SD.

Secondly, we examined whether PDGF-BB, an intense growth factor of PASMCs, up-regulated the expression of RAGE. Western blot analysis revealed that PDGF-BB (10 ng/mL) up-regulated the expression of RAGE in IPAH, HPAH and non-PAH-PASMCs (Fig 3A–3C).

### Inhibitory effects of AS-1 on proliferation of PAH-PASMCs

**Fig 4:**
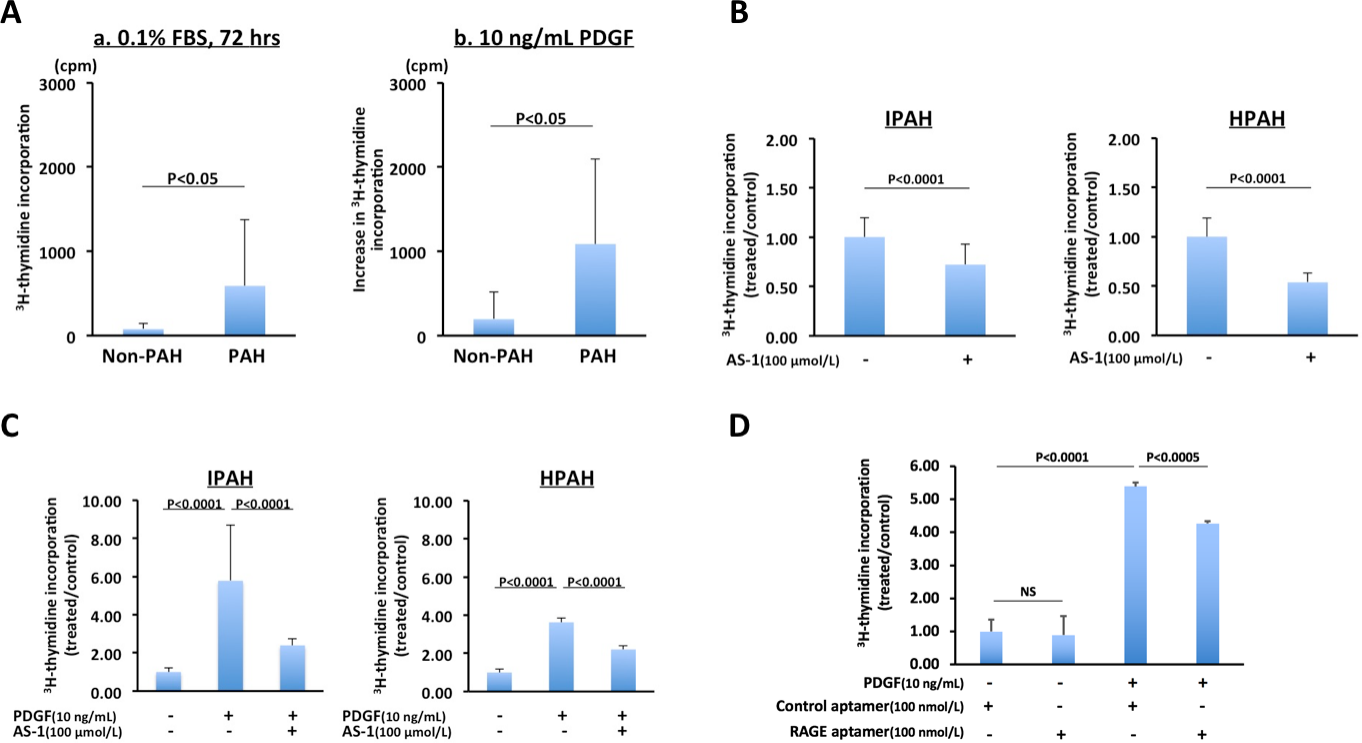
Inhibitory effects of AS-1 or RAGE aptamer on proliferation of PAH-PASMCs assessed by ^3^H-thymidine incorporation. A. Comparison of proliferation of PASMCs from non-PAH patients with that of PASMCs from PAH patients under a condition of no growth simulation (left) and PDGF-BB (10 ng/mL)-stimulated condition (right). B. AS-1, an inhibitor of TIR domain-mediated RAGE signaling, significantly inhibited overgrowth under a condition of no growth simulation in IPAH- (left) and HPAH-PASMCs (right). C. AS-1 significantly inhibited PDGF-stimulated proliferation of IPAH- (left) and HPAH-PASMCs (right). D. DNA aptamer significantly inhibited PDGF-stimulated proliferation of a HPAH patient with BMPR2 mutation. Data are mean ± SD.

Next, we compared proliferation of PASMCs from non-PAH patients with that of PASMCs from PAH patients. PAH-PASMCs were hyperplastic in the absence of any external growth stimulus including PDGF as assessed by ^3^H-thymidine incorporation (Fig 4A, panel a). This result indicates abnormal proliferation and overgrowth characterized by continued growth under a condition of no growth stimulation in PAH-PASMCs. Moreover, PDGF-BB (10 ng/mL) stimulation caused a higher growth rate of PAH-PASMCs than that of non-PAH-PASMCs (Fig 4A, panel b).

Next, we examined whether AS-1, an inhibitor of RAGE signaling, has anti-proliferative effects on PAH-PASMCs. AS-1 significantly inhibited overgrowth under a condition of no growth stimulation in IPAH and HPAH-PASMCs (Fig 4B). Furthermore, AS-1 significantly inhibited PDGF-stimulated proliferation of IPAH and HPAH-PASMCs (Fig 4C).

Recently, we reported that a DNA aptamer, a short and single-stranded DNA, directed against RAGE (RAGE aptamer) inhibited the binding of AGE to RAGE and attenuated the development and progression of streptozotocin-induced diabetic nephropathy in rats and deoxycorticosterone acetate/salt-induced renal injury in mice [17, 18]. We examined the inhibitory effects of the RAGE aptamer on PDGF-induced proliferation of PASMCs. The RAGE aptamer significantly inhibited PDGF-stimulated proliferation of PASMCs from a HPAH patient with BMPR2 mutation (patient number 12) (n = 8 experiments) (Fig 4D).

To further assess the link between expression of RAGE and proliferation of PASMCs, we investigated gene expression in PDGF-BB-stimulated IPAH-PASMCs before and after AS-1 treatment using Human EGF/PDGF Signaling Pathway PCR Array (SABiosciences, a QIAGEN company). PCR-array analysis showed that AS-1 down-regulated mitogen-activated protein kinase 3 (*MAPK3*) gene expression in PDGF-BB-stimulated IPAH-PASMCs (S1 File). Further studies are needed to clarify this point.

## Discussion

Three major findings were obtained in the present study. First, RAGE, S100A8 and S100A9 were overexpressed in IPAH and HPAH-PASMCs in the absence of any external growth stimulus. Second, PDGF-BB up-regulated the expression of RAGE in IPAH and HPAH-PASMCs. Third, AS-1, an inhibitor of RAGE signaling, significantly inhibited overgrowth under a condition of no growth stimulation and PDGF-stimulated proliferation of IPAH and HPAH-PASMCs. RAGE plays an important role in the inappropriate increase of PAH-PASMCs, and its inhibition may be a new therapeutic strategy for PAH.

Several investigators have reported that expression levels of RAGE were increased in serum or plasma, small pulmonary arteries and PASMCs of patients with PAH [9, 19, 20]. Here, we showed that RAGE was overexpressed in PASMCs of not only patients with IPAH but also patients with HPAH including patients with *BMPR2* mutation and *SMAD9* mutation. Therefore, RAGE is a common exacerbating factor in PAH. As for ligands for RAGE, Greenway et al. reported that S100A4/Mts1 was expressed in smooth muscle cells of lesions showing neointimal formation and with increased intensity in vessels with an occlusive neointima and plexiform lesions of patients with pulmonary hypertension secondary to congenital heart disease [21]. Meloche et al. reported that activation of RAGE by S100A4 decreased BMPR2 expression in human PASMCs [9]. We showed that S100A8 and A9 were also overexpressed in IPAH and HPAH-PASMCs. We previously reported that S100A8/A9 induced phosphorylation of RAGE and led to activation of diverse signal effectors such as Rac1, Akt, p38, JNK, IKK, and NFkB in HEK293 and 293-hMD2-CD14 cells [6]. Further studies are needed to clarify the precise role of S100A8 and A9 in the pathogenesis of PAH.

We previously reported that PDGF-BB stimulation caused a higher growth rate of cultured PASMCs from patients with IPAH than that of control cells [3, 10]. Here we showed that PDGF-BB also up-regulated the expression of RAGE in IPAH and HPAH-PASMCs.

We have recently reported that Toll/IL-1 receptor (TIR) domain-containing adaptor protein (TIRAP) transduces RAGE signaling and that functional interaction between RAGE and TLRs coordinately regulates inflammation, immune response and other cellular functions [6]. A synthetic low-molecular-weight TIR/BB-Loop mimetic, AS-1, inhibits TIR domain-mediated signaling [22]. AS-1 finally inhibits RAGE signaling. AS-1, an inhibitor of RAGE signaling, significantly inhibited overgrowth under a condition of no growth stimulation and PDGF-stimulated proliferation of IPAH and HPAH-PASMCs.

This study was performed in a small population of patients. Therefore, we could not perform quantitative analysis of staining. This is a study limitation. Further study is needed.

In conclusion, RAGE plays a crucial role in the inappropriate increase of PAH-PASMCs. Inhibition of RAGE signaling may be a new therapeutic strategy for PAH.

## Supporting information

S1 File Human EGF/PDGF signaling pathway PCR Array in PDGF-BB-stimulated IPAH-PASMCs before and after AS-1 treatment. (DOCX)
