# Supplementary material for: Crucial role of RAGE in inappropriate increase of smooth muscle cells from patients with pulmonary arterial hypertension
Source: PLoS One. 2018 Sep 4;13(9):e0203046. doi: 10.1371/journal.pone.0203046 (PMC6122782; doi:10.1371/journal.pone.0203046)
Supplement: S1 File — (DOCX) [file pone.0203046.s001.docx]

**S1. Human EGF/PDGF signaling pathway PCR Array in PDGF-BB-stimulated IPAH-PASMCs before and after AS-1 treatment**

**Methods**

Human EGF/PDGF signaling pathway PCR Array in PDGF-BB-stimulated IPAH-PASMCs before and after AS-1 treatment was performed with the RT^2^ Profiler PCR Array System (SABiosciences, a QIAGEN company) according to the manufacturer’s instructions. The array measures 84 key genes involved in EGF/PDGF signaling pathway. Total RNA from PASMCs of a patient with IPAH was extracted using RNeasy Mini Kit (QIAGEN). Complementary DNA was synthesized from 1 μg of total RNA using ReverTra Ace^®^ (Toyobo Life Science, Tokyo) as prescribed in the manual and subjected to PCR amplification. Expression of mRNA was measured by reverse transcription PCR (RT-PCR) using an ABI PRISM 7300 sequence detector system (Applied Biosystems).

**Results**

| **S1 Table. EGF/PDGF signaling pathway PCR Array in PDGF-BB-stimulated IPAH-PASMCs before and after AS-1 treatment** | | |
| --- | --- | --- |
| **Gene Symbol** | **Gene Description** | **Fold Regulation** |
| ACTR2 | ARP2 actin-related protein 2 homolog (yeast) | 1.7305 |
| AKT1 | V-akt murine thymoma viral oncogene homolog 1 | 1.5391 |
| AKT2 | V-akt murine thymoma viral oncogene homolog 2 | -5.0704 |
| AKT3 | V-akt murine thymoma viral oncogene homolog 3 (protein kinase B, gamma) | 1.1057 |
| ARAF | V-raf murine sarcoma 3611 viral oncogene homolog | 1.0051 |
| ATF1 | Activating transcription factor 1 | 1.9641 |
| ATF2 | Activating transcription factor 2 | 1.1323 |
| BAD | BCL2-associated agonist of cell death | -1.3415 |
| BCAR1 | Breast cancer anti-estrogen resistance 1 | -1.2932 |
| BCL2 | B-cell CLL/lymphoma 2 | -1.5476 |
| BRAF | V-raf murine sarcoma viral oncogene homolog B1 | 1.5943 |
| CASP3 | Caspase 3, apoptosis-related cysteine peptidase | 1.3295 |
| CASP9 | Caspase 9, apoptosis-related cysteine peptidase | 1.0658 |
| CBL | Cas-Br-M (murine) ecotropic retroviral transforming sequence | -1.1261 |
| CCND1 | Cyclin D1 | 1.2705 |
| CHUK | Conserved helix-loop-helix ubiquitous kinase | 1.4388 |
| COL1A1 | Collagen, type I, alpha 1 | 2.7725 |
| CREB1 | CAMP responsive element binding protein 1 | 4.1305 |
| CSNK2A1 | Casein kinase 2, alpha 1 polypeptide | 2.2044 |
| CSNK2B | Casein kinase 2, beta polypeptide | 1.0849 |
| DUSP1 | Dual specificity phosphatase 1 | -1.0506 |
| DUSP6 | Dual specificity phosphatase 6 | 2.3545 |
| EGF | Epidermal growth factor | 2.6405 |
| EGFR | Epidermal growth factor receptor | 1.1822 |
| EGR1 | Early growth response 1 | -1.8896 |
| EIF4E | Eukaryotic translation initiation factor 4E | 4.4079 |
| ELK1 | ELK1, member of ETS oncogene family | -3.4075 |
| EPS8 | Epidermal growth factor receptor pathway substrate 8 | 1.0937 |
| FASLG | Fas ligand (TNF superfamily, member 6) | -1.2592 |
| FN1 | Fibronectin 1 | 1.1108 |
| FOS | FBJ murine osteosarcoma viral oncogene homolog | -3.5983 |
| FOXO3 | Forkhead box O3 | 1.4066 |
| GAB1 | GRB2-associated binding protein 1 | -1.1495 |
| GRB2 | Growth factor receptor-bound protein 2 | -1.1265 |
| GSK3A | Glycogen synthase kinase 3 alpha | 1.0376 |
| GSK3B | Glycogen synthase kinase 3 beta | 1.6909 |
| HBEGF | Heparin-binding EGF-like growth factor | 1.4743 |
| HRAS | V-Ha-ras Harvey rat sarcoma viral oncogene homolog | -1.2271 |
| IKBKB | Inhibitor of kappa light polypeptide gene enhancer in B-cells, kinase beta | -1.6119 |
| IL2 | Interleukin 2 | -1.2592 |
| JAK1 | Janus kinase 1 | -1.016 |
| JUN | Jun proto-oncogene | 1.0207 |
| KRAS | V-Ki-ras2 Kirsten rat sarcoma viral oncogene homolog | 1.5505 |
| LTA | Lymphotoxin alpha (TNF superfamily, member 1) | -1.6699 |
| MAP2K1 | Mitogen-activated protein kinase kinase 1 | 4.1583 |
| MAP2K4 | Mitogen-activated protein kinase kinase 4 | -1.0977 |
| MAP2K7 | Mitogen-activated protein kinase kinase 7 | 1.1504 |
| MAP3K2 | Mitogen-activated protein kinase kinase kinase 2 | 1.4699 |
| MAPK1 | Mitogen-activated protein kinase 1 | 1.1642 |
| MAPK10 | Mitogen-activated protein kinase 10 | -1.4145 |
| MAPK3 | Mitogen-activated protein kinase 3 | -9.4146 |
| MAPK8 | Mitogen-activated protein kinase 8 | 1.105 |
| MAPK9 | Mitogen-activated protein kinase 9 | 1.4099 |
| MKNK1 | MAP kinase interacting serine/threonine kinase 1 | -1.0758 |
| MMP7 | Matrix metallopeptidase 7 (matrilysin, uterine) | -1.87 |
| NCK2 | NCK adaptor protein 2 | -1.1212 |
| NFATC3 | Nuclear factor of activated T-cells, cytoplasmic, calcineurin-dependent 3 | 1.7674 |
| NFKB1 | Nuclear factor of kappa light polypeptide gene enhancer in B-cells 1 | 1.264 |
| NRAS | Neuroblastoma RAS viral (v-ras) oncogene homolog | 1.2529 |
| NUP62 | Nucleoporin 62kDa | 1.58 |
| PDGFA | Platelet-derived growth factor alpha polypeptide | -1.5633 |
| PDGFB | Platelet-derived growth factor beta polypeptide | -2.1615 |
| PDGFRA | Platelet-derived growth factor receptor, alpha polypeptide | -1.9961 |
| PDPK1 | 3-phosphoinositide dependent protein kinase-1 | -1.0403 |
| PIK3CA | Phosphoinositide-3-kinase, catalytic, alpha polypeptide | 2.501 |
| PIK3R1 | Phosphoinositide-3-kinase, regulatory subunit 1 (alpha) | 1.0173 |
| PIK3R2 | Phosphoinositide-3-kinase, regulatory subunit 2 (beta) | -1.4316 |
| PLAT | Plasminogen activator, tissue | 1.2359 |
| PLCG1 | Phospholipase C, gamma 1 | -1.2488 |
| PPP2CA | Protein phosphatase 2, catalytic subunit, alpha isozyme | 1.5301 |
| PRKCA | Protein kinase C, alpha | 1.4388 |
| PTEN | Phosphatase and tensin homolog | 1.108 |
| RAF1 | V-raf-1 murine leukemia viral oncogene homolog 1 | -1.3474 |
| RAP1A | RAP1A, member of RAS oncogene family | 1.018 |
| RASA1 | RAS p21 protein activator (GTPase activating protein) 1 | 1.0949 |
| RHOA | Ras homolog gene family, member A | -1.213 |
| RPS6KA5 | Ribosomal protein S6 kinase, 90kDa, polypeptide 5 | -1.0018 |
| RPS6KB1 | Ribosomal protein S6 kinase, 70kDa, polypeptide 1 | 1.7293 |
| SHC1 | SHC (Src homology 2 domain containing) transforming protein 1 | 1.0583 |
| SRC | V-src sarcoma (Schmidt-Ruppin A-2) viral oncogene homolog (avian) | -1.1726 |
| STAT1 | Signal transducer and activator of transcription 1, 91kDa | -1.2754 |
| STAT3 | Signal transducer and activator of transcription 3 (acute-phase response factor) | 1.1104 |
| STAT5A | Signal transducer and activator of transcription 5A | -1.1611 |
| TP53 | Tumor protein p53 | 3.7934 |
| B2M | Beta-2-microglobulin | 1.023 |
| HPRT1 | Hypoxanthine phosphoribosyltransferase 1 | 1.82 |
| RPL13A | Ribosomal protein L13a | -3.4271 |
| GAPDH | Glyceraldehyde-3-phosphate dehydrogenase | 1 |
| ACTB | Actin, beta | 3.0314 |
